# Profiles of Proinflammatory Cytokines and T Cells in Patients With Tourette Syndrome: A Meta-Analysis

**DOI:** 10.3389/fimmu.2022.843247

**Published:** 2022-05-26

**Authors:** Ying Li, Xiaolin Wang, Hanxue Yang, Yanlin Li, Jingang Gui, Yonghua Cui

**Affiliations:** ^1^Department of Psychiatry, Beijing Children’s Hospital, Capital Medical University, National Centre for Children’s Health, Beijing, China; ^2^Laboratory of Tumor Immunology, Beijing Pediatric Research Institute, Beijing Children’s Hospital, Capital Medical University, National Center for Children’s Health, Beijing, China; ^3^Neuropsychology and Applied Cognitive Neuroscience Laboratory, Institute of Psychology, Chinese Academy of Sciences, Beijing, China; ^4^Chinese Academy of Sciences (CAS) Key Laboratory of Mental Health, Institute of Psychology, Beijing, China

**Keywords:** Tourette syndrome, proinflammatory cytokines, T cell, immunological dysfunction, meta-analysis

## Abstract

**Background:**

Tic disorder is a neurodevelopmental disorder characterized by motor and phonic tic symptoms. Tourette syndrome (TS) is a subtype of tic disorder that shows more persistent tic symptoms. The etiological mechanism of TS concerning immune dysfunction remains unclear due to limited evidence, especially for pediatric TS patients.

**Method:**

In the present study, a meta-analysis was performed to confirm the identified changes in proinflammatory cytokines and T cells of pediatric TS patients. A total of five databases, including PubMed, Web of Science, PsycINFO, Google Scholar and the China National Knowledge Infrastructure (CNKI), were used for the literature search. The standardized mean difference (SMD) and mean difference (MD) with a 95% confidence interval (CI) were used to present the effect size of each type of proinflammatory cytokine and T cell. Sensitivity analysis, subgroup analysis and meta-regression analysis were used to explore the heterogeneity of the meta-analysis. This meta-analysis was registered in the International Platform of Registered Systematic Review and Meta-analysis Protocols (number: INPLASY2021110079).

**Results:**

In the 25 studies included in this meta-analysis, thirteen studies focused on the levels of T cells, and twelve studies focused on the levels of proinflammatory cytokines. Based on the random-effects model, the pooled MDs are -1.45 (95% CI: -3.44, 0.54) for CD3 cells, -4.44 (95% CI: -6.80, -2.08) for CD4 cells, and 1.94 (95% CI: -0.08, 3.97) for CD8 cells. The pooled SMDs are1.36 for IL-6 (95% CI: 0.00, 2.72) and 2.39 for tumor necrosis factor alpha (TNF-α) (95% CI: 0.93, 3.84).

**Conclusion:**

We provided evidence of immune dysfunction in pediatric TS patients, with elevated levels of particular proinflammatory cytokines and disproportionate changes in T-cell subpopulations. Small to large effect sizes were identified for increased IL-6 levels as well as a reduced number of T helper cells, while a large effect size was identified for increased TNF-α levels. These results indicate a close association between peripheral immune activation and TS. However, the most direct and meaningful interaction between peripheral immune status and microglial activation in the central nervous system in TS patients requires further exploration.

## Introduction

Tic disorders (TDs) are common neurodevelopmental disorders in children and adolescents. According to the American Psychiatric Association’s Diagnostic and Statistical Manual of Mental Disorders, Fifth Edition (DSM-5), Tourette’s syndrome (TS), chronic motor tic disorder (CMTD), chronic vocal tic disorder (CVTD), and transient tic disorder (TTD) are the main diagnostic types of TDs (1). Of them, TS shows more persistent tic symptoms (2), and patients with TS commonly have poorer prognosis than those with other types of tic disorders (3). According to a previous investigation, TS affects approximately 4 to 8 per 1000 children and is associated with hyperactivity, impulsiveness, inattention and emotional problems (4). Despite numerous attempts to clarify the pathophysiology of TS from behavioral and brain imaging levels (5, 6) to genetic and immunological levels (7, 8), the etiology of TS is still not well established (9). It is worth noting that immune dysfunction has been regarded as one of the most important factors involved in the onset and development of TS (10).

Tic symptoms generally start at the age of 7-8 years and reach their utmost severity at approximately 8-12 years old (11, 12), with occasional reoccurrences throughout the lifespan in many cases (13). Accumulated evidence has suggested that relapse of tic symptoms during the long pathophysiological course of TS might be triggered by improper immune activation and inflammation (14, 15). A larger number of TS patients were identified to have group A streptococcal (GAS) infection that was not in the healthy group (16). Moreover, a group of TS patients have also been proven to have streptococcal infection (17). Other infections, such as mycoplasma and enterovirus (EV), are also reported to be associated with tic symptoms (10, 18, 19). Infections commonly trigger immune activation and proinflammatory reactions. For instance, it has been documented that GAS infection in TS patients induced an increased serum antistreptolysin O (ASO) level (7). However, the most recent study by the European multicenter tics in children identified that GAS exposure is not associated with the development of tics in children with a chronic tic disorder (20). CD69^+^ B lymphocytes and CD95^+^ T lymphocytes have been revealed to be markedly increased in adult TS patients (21). It has been commonly believed that a skewed increase in particular proinflammatory cytokines as well as a deviated change in a particular T-cell component are closely associated with TS (10).

Recently, only a narrative review (10) and a meta-analysis on proinflammatory cytokines in TS have been reported (7). Notably, most present studies on the inflammatory environment in TS are limited by small sample sizes (10). However, the conclusion of that meta-analysis is limited by the very small number of enrolled studies (only 2-3 studies were included in each meta-analysis). Therefore, a comprehensive meta-analysis with more exhaustive inclusion of TS-related studies is warranted to determine the relationship between immune dysfunction and the disease progression of TS. To achieve this aim, we conducted a meta-analysis to delineate the profiles of immune cells and proinflammatory cytokines in patients with TS. Our analysis based on the results of previous studies indicated that deviation in the T-cell compartment and proinflammatory cytokines in peripheral circulation are featured in TS patients.

## Materials and Methods

### Literature Search

A systematic search was performed in the PubMed, Elsevier, and China National Knowledge Infrastructure (CNKI) databases. The keywords used to identify studies are as follows: (‘tic’ or ‘Tourette’ or ‘TD’ or ‘TS’ or ‘Tourette syndrome’) and (‘cytokines’ or ‘TNF-α’ or ‘IL-2’ or ‘IL-4’ or ‘IL-6’ or ‘IL-8’ or ‘IL-12’ or ‘IFN-γ’ or ‘CD3’ or ‘CD4’ or ‘CD8’ or ‘CD4/CD8’ or ‘T-cell’). When searching the CNKI database, we used the corresponding formal translation terms (in Chinese) mentioned above. The included studies (up to 31 October 2021) subjected to our meta-analysis were independently cross-checked by two researchers to verify their relevance to the topic. This study is registered in the International Platform of Registered Systematic Review and Meta-analysis Protocols (INPLASY, number: INPLASY2021110079).

### Inclusion and Exclusion Criteria

To identify relevant studies for our meta-analysis, we developed the following inclusion and exclusion criteria:

The inclusion criteria are as follows:

(1) English or Chinese studies from peer-reviewed journals.
(2) The included patients are diagnosed with Tourette syndrome-related disorders.
(3) T cells and cytokines are assessed in the serum or plasma of peripheral blood.

The exclusion criteria are as follows:

(1) Case reports, reviews, or meta-analyses.
(2) Studies with a sample size less than 5.
(3) Studies involving rats or mice, rather than humans.
(4) Studies that included the same participants in the included studies.

### Quality Assessment for the Included Studies

The quality of each study is assessed by the modified Critical Appraisal Skills Programme (CASP) scale. The CASP tool is widely used for appraising the limitations and strengths of any qualitative research methodology (22, 23). This tool included 11 items such as ‘Item 1: Did the study address a clearly focused issue?’ or ‘Item 4: Were the controls selected in an acceptable way?’. Studies were chosen or discarded until a consensus was reached after independent assessment from two authors. Studies are excluded when less than 6 ‘Yes’ responses after CASP scale processing.

### Data Extraction

The following information was extracted from the included studies: authors, publication years, countries, sample sizes (patients/controls), mean ages (years), types of proinflammatory cytokines and T cells, Yale Global Tic Severity Scale (YGTSS) scores (mean ± standard deviation), and techniques for measuring the T cells/proinflammatory cytokines.

### Statistical Analysis

I^2^ statistics and forest plots are used to identify the heterogeneity of the meta-analysis. Provided that I^2^ was greater than 50%, a random-effects model is applied (24). Egger’s test is employed to judge whether there is publication bias. A sensitivity analysis is also performed to identify the study with high heterogeneity (omitting one study at a time and tracking the change in I^2^ to identify the contribution of each study to the heterogeneity) (25). The standardized mean difference (SMD) is calculated to measure the effect size in each included study (for the SMD calculation formula, please see **Supplementary Figure 1**). An SMD value between 0.2 and 0.5 is considered mild-to-moderate, whereas an SMD value between 0.5 and 0.8 indicates that the efficacy is moderate-to-large (26). Moreover, the mean difference (MD) is also used to calculate the effect size of the meta-analysis. If the included studies are based on the same sample and same technique, the MD is used; if not, the SMD is used. We consider a *p* value < 0.05 to be statistically significant, and all the analyses are performed in R (version 3.5.3) using the “meta” or “metafor” package (27).

## Results

### The Description of the Included Studies

**Figure 1:**
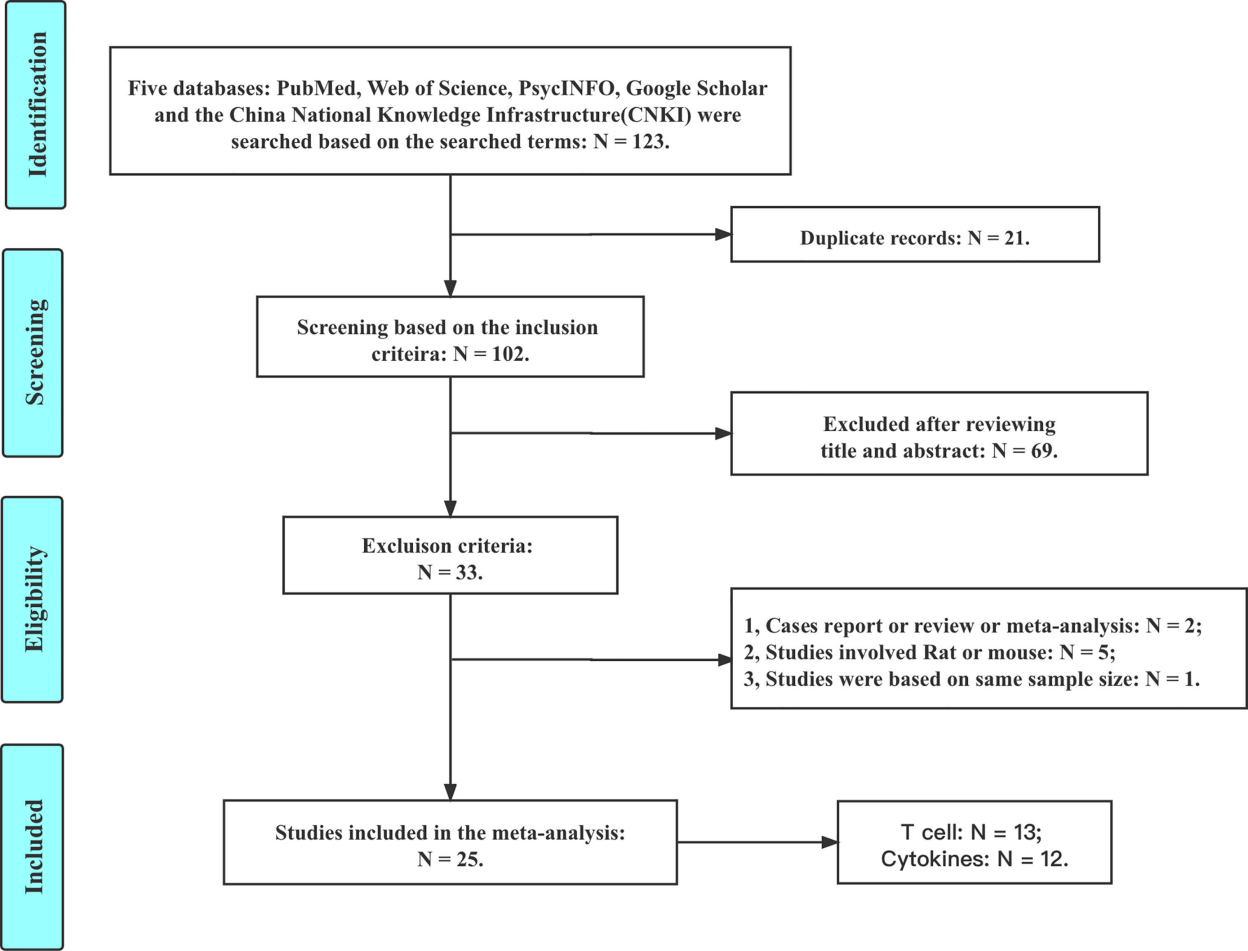
Flowchart of the selection criteria.

Based on the inclusion and exclusion criteria, a total of 25 studies are included in this meta-analysis (details are shown in **Figure 1**). There are 13 studies reporting the CD3, CD4, and CD8 levels; 4 studies reporting the IL-2 level; 4 studies reporting the IL-4 level; 7 studies reporting the IL-6 level; 4 studies reporting the IL-8 level; 5 studies reporting the IL-12 level; 4 studies reporting the INF-γ level; and 8 studies reporting the TNF-α level.

We summarize the expression of several proinflammatory cytokines and T cells in TS patients and healthy controls. All included studies measure cytokine levels in serum or plasma from participants with TS. The expression of seven cytokines (TNF-α, IL-2, IL-4, IL-6, IL-8, IL-12 and IFN-γ) and the proportions of T cells (CD3, CD4, CD8) are summarized. Furthermore, we list the authors, publication years, countries, sample sizes (patients/controls), mean ages (years), types of proinflammatory cytokines and T cells, YGTSS scores (mean ± standard deviation) and the techniques used in each included study (**Table 1**).

**Table 1:** The included studies for this meta-analysis.

Study	Year	Country	Patient/Control	Age (years)	YGTSS score	Sample	Cell types/Cytokines	Technique
Zeynep et al. (28)	2021	Turkey	48/24	11.6/11.6	–	peripheral blood	CD3, CD4, CD8 T cells	flow cytometry
Ma (29)	2021	China	80/80	8.9/9.0	–	serum	TNF-α, IL-2, IL-6, IL-8	ELISA
Liu (30)	2020	China	100/78	8.8/9.6	–	serum	IL-8	ELISA
Hou^a^ et al. (31)	2018	China	150/80	7.5/7.6	–	peripheral blood	CD3, CD4, CD8 T cells	flow cytometry
He (32)	2018	China	66/38	8.8/9.1	–	peripheral blood	CD3, CD4, CD8 T cells	flow cytometry
Pranzatelli et al. (33)	2017	USA	5/26	10/-	50±31	peripheral blood	CD3, CD4, CD8 T cells	flow cytometry
Lu et al. (34)	2017	China	21/30	9.8/10.1	–	peripheral blood	CD3, CD4, CD8 T cells	flow cytometry
Fan et al. (35)	2017	China	74/20	7.9/9.0	–	serum	TNF-α, IL-6	ELISA
Chen et al. (36)	2016	China	40/40	7.9/7.2	–	peripheral blood	CD3, CD4, CD8 T cells	flow cytometry
Cheng et al. (37)	2016	China	52/52	-/-	–	peripheral blood, serum	CD3, CD4, CD8 T cells, TNF-α, IL-6, IL-8	flow cytometry, ELISA
Gao et al. (38)	2016	China	40/40	8.8/8.2	65.31±9.85	serum	IFN-γ, IL-4	ELISA
Erzhen at al. (39)	2015	China	58/45	9.7/8.9	31.18±6.70	peripheral blood	CD3, CD4, CD8 T cells	flow cytometry
Zhang et al. (40)	2015	China	31/30	9.0/8.0	–	peripheral blood	CD3, CD4, CD8 T cells	flow cytometry
Zhang et al. (41)	2014	China	41/60	10.0/10.0	–	serum	IFN-γ, IL-12, IL-2, IL-4	ELISA
Tang et al. (42)	2014	China	30/30	10.7/10.8	16.14±6.94	serum	TNF-α, IL-12	ELISA
Luo et al. (43)	2014	China	40/24	7.7/8.1	–	serum	TNF-α, IL-2	ELISA
Liu et al. (44)	2013	China	57/43	9.7/9.4	–	peripheral blood	CD3, CD4, CD8 T cells	flow cytometry
Li et al. (45)	2013	China	32/30	10.1/10.7	–	peripheral blood, serum	CD3, CD4, CD8 T cells, IL-6, IL-8	flow cytometry, ELISA
Yu-hang et al. (46)	2012	China	40/40	13.0/12.4	–	plasma	IL-6	ELISA
Ji (47)	2011	China	33/30	10.0/9.6	–	peripheral blood	CD3, CD4, CD8 T cells	flow cytometry
Gabbay et al. (48)	2009	USA	32/16	11.2/15.1	22.0±6.11	plasma	TNF-α, IL-12, IL-6	ELISA
Zhang (49)	2008	China	30/30	10.1/10.5	–	peripheral blood	CD3, CD4, CD8 T cells	flow cytometry
Mao (50)	2008	China	25/15		–	serum	TNF-α, IL-12	ELISA
Leckman et al. (51)	2005	USA	46/31	11.8/12.5	–	serum	IFN-γ, IL-12, TNF-α, IL-2, IL-4, IL-6	ELISA
***Hou et al. ^b^*** (31)	***2018***	***China***	***40/40***	***7.5/7.6***	***-***	***serum***	***IFN-γ, IL-4***	***ELISA***

YGTSS, Yale Global Tic Severity Scale; IL, Interleukin; TNF-α, tumor necrosis factor alpha; IFN-γ, Interferon gamma; ELISA, the enzyme-linked immunosorbent assay; Hou XJa and Hou XJb were from the same study but different sample. Bold vaule means same study with different sample.

### Quality Assessment and Publication Bias for the Included Studies

Assessments of the CASP scale for each included study are shown in **Supplementary Table 1** (all included studies met the criteria for quality assessment). Egger’s test values, the degree of the test, and the *p* value are shown in **Table 2**. No publication bias is identified for the meta-analysis of CD3, CD4, CD8, IL-2, IL-4, IL-6, IL-8, IL-12 and TNF-α (*p* > 0.05). However, significant publication bias is identified in the meta-analysis of IFN-γ (*p* = 0.02). Therefore, the included studies for IFN-γ are not suitable for the meta-analysis. Details are shown in **Table 2**.

**Table 2:** The publication bias by Egger test.

Meta-analysis	Number of included studies	T value	df	p-value
IL-2	4	1.67	2	0.24
IL-4	4	-1.05	2	0.40
IL-6	7	1.60	5	0.17
IL-8	4	2.75	2	0.11
IL-12	5	3.08	3	0.05 (0.0542)
TNF-α	8	1.65	6	0.15
IFN-γ	4	6.44	2	0.02*
CD3	13	-0.60	11	0.56
CD4	13	-0.23	11	0.82
CD8	13	-0.65	11	0.53
CD4/CD8	11	-0.03	9	0.97

*P<0.05.

### Meta-Analysis of T Cells

**Figure 2:**
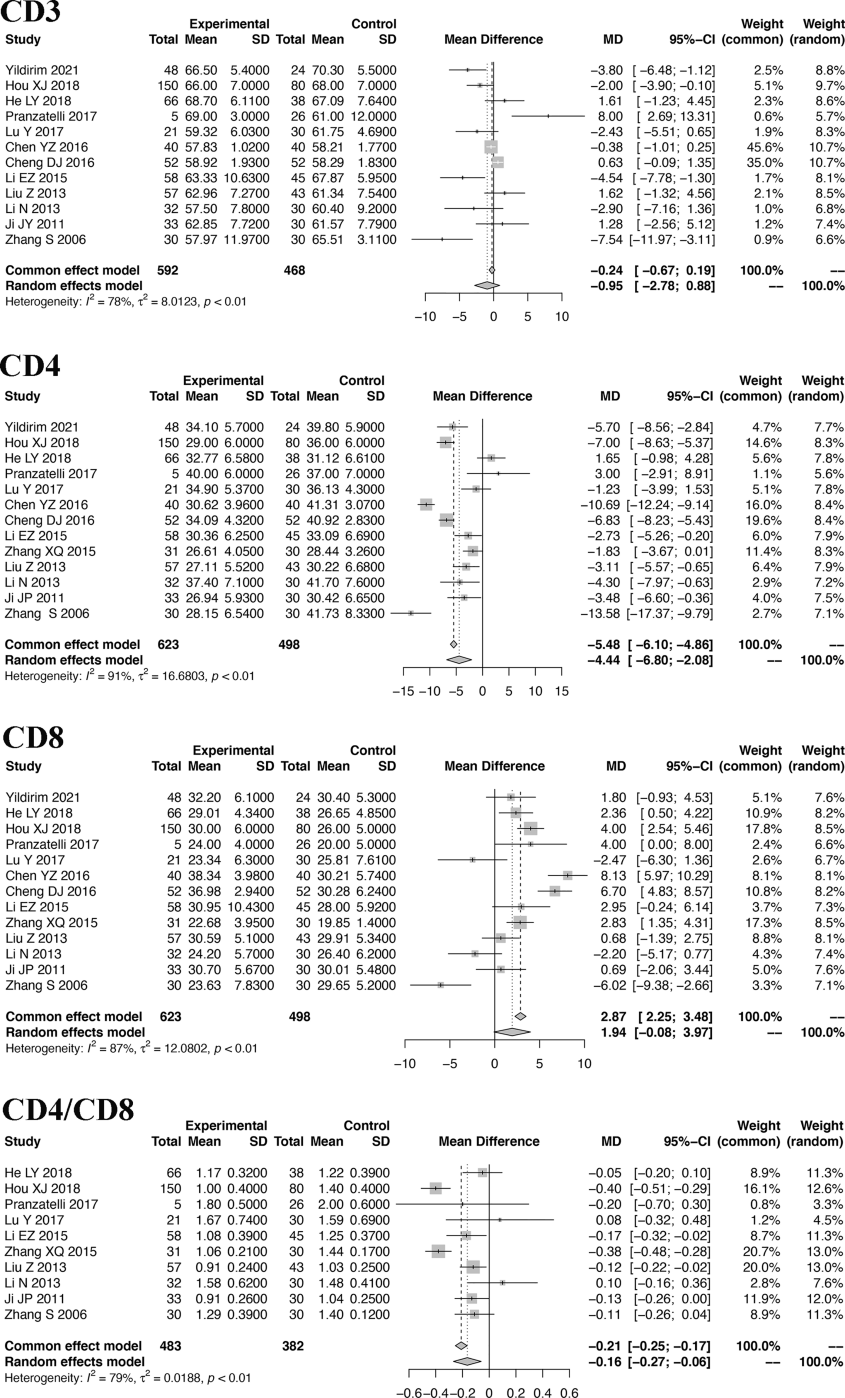
Forest plot of the meta-analysis of T cells.

Because the included studies of T cells are based on the same types of samples and the same technique, the MD was used to show the pooled effect size of T cells. The pooled MDs of CD3, CD4, CD8 and CD4/CD8 cells with 95% confidence intervals (CIs) were calculated. Based on the random-effects model, the pooled MDs are -1.45 (95% CI: -3.44, 0.54) for CD3 cells, -4.44 (95% CI: -6.80, -2.08) for CD4 cells, 1.94 (95% CI: -0.08, 3.97) for CD8 cells, and -0.20 (95% CI: -0.32, -0.08) for CD4/CD8 cells. Details are shown in **Figure 2**.

### Meta-Analysis of Proinflammatory Cytokines

**Figure 3:**
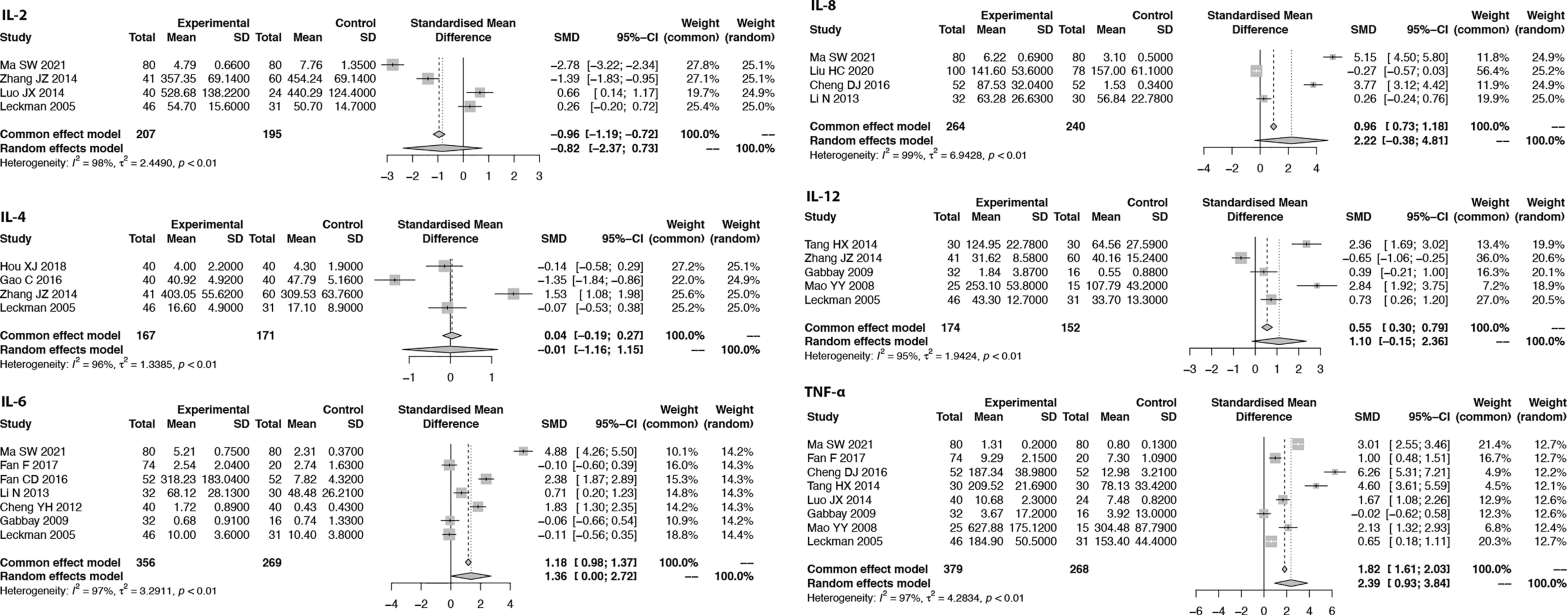
Forest plots of the meta-analysis of proinflammatory cytokines.

Due to the measured cytokine concentrations derived from different sample sources (plasma and serum) in the included studies, the pooled SMD with 95% CIs is used to assess the effect size of proinflammatory cytokines. When the I^2^ of the pooled SMD is more than 50%, the random-effects model is selected. We found that the pooled SMD is 0.82 (95% CI: -2.37, 0.73) for IL-2, -0.01 (95% CI: -1.16, 1.15) for IL-4, 1.36 (95% CI: 0.00, 2.72) for IL-6, 2.22 (95% CI: -0.38, 4.81) for IL-8, 1.10 (95% CI: -0.15, 2.36) for IL-12, and 2.39 (95% CI: 0.93, 3.84) for TNF-α (**Figure 3**). Since publication bias was detected for IFN-γ, we presented the results for IFN-γ in **Supplementary Figure 2** as evidence of lower grade.

### Sensitivity Analysis of the Meta-Analysis

Sensitivity analysis is performed to explore the heterogeneity of the pooled SMD. The results indicate that there are no studies with I^2^ changes greater than 5% in the meta-analysis of IL-2, IL-6, IL-8, IL-12, TNF-α, CD4, and CD8. However, in the sensitivity analysis of IL-4, one study might have increased the heterogeneity (with I^2^ changes greater than 5%) (41). When we exclude this study, the modified pooled SMD for IL-4 is -0.52 (95% CI: -1.32, 0.29). In the sensitivity analysis of CD3, one study might have increased the heterogeneity (40). After the exclusion of this study, the modified pooled MD for CD3 cells is -0.95 (95% CI: -2.77, 0.88).

### Subgroup Analysis and Meta-Regression Analysis

Subgroup analysis by ‘Mean Age’ (Group A below 10 years old, Group B above 10 years old) is performed in the meta-analysis of TNF-α and IL-6 (the number of included studies for these two studies is more than 5). However, no significant difference was identified in the test for the subgroup differences (random effect model) of IL-6 (*p* = 0.24) and TNF-α (*p* = 0.57) (**Supplementary Figure 3**).

Meta-regression analysis of ‘Mean Age’ and ‘Publication Year’ for CD4 and CD8 was performed, and the results of the meta-regression analysis are summarized in **Table 3**. Only the ‘Publication Year’ for CD8 was significant (accounting for 31.80% of the heterogeneity, *p* = 0.02).

**Table 3:** The meta-regression analysis for the Mean Age and Publication Year to CD4 and CD8.

Predictors	Number of included studies	Tau^2^	I^2^	H^2^	R^2^	QM	P value
Publication Year to CD4	13	15.50	92.10%	12.65	7.10%	2.19	0.14
Publication Year to CD8	13	8.24	86.23%	7.26	31.80%	5.61	0.02^*^
Mean Age to CD4	13	18.25	92.65%	13.60	0.00%	0.15	0.70
Mean Age to CD8	13	11.01	89.15%	9.21	8.87%	2.02	0.16

*P < 0.05; Tau^2^, estimated amount of residual heterogeneity; I^2^, residual heterogeneity or unaccounted variability; H2, unaccounted variability / sampling variability; R^2^, amount of heterogeneity accounted for; QM: the statistic of the test of predictors.

### Other Immunological Indicators Associated With Tourette Syndrome

In addition to T cells and peripheral proinflammatory cytokines, we also searched for other immunological indicators associated with Tourette syndrome. The data for the first author, publication year, sample size, mean and standard deviation of these immunological indicators in the experimental group and the control group are listed in **Table 2** as the **Supplementary Materials**. We found equivocal results in immunoglobulin- and B-cell-related studies on Tourette syndrome. Of note, one study reported increased monocytes/macrophages in Tourette syndrome patients, while the fact that only one publication mentioned the change in monocytes/macrophages made us unable to perform any meaningful meta-analysis (52).

## Discussion

In the present study, our results indicate that the levels of proinflammatory cytokines are increased in pediatric patients with Tourette syndrome. Increased CD4 T-cell and decreased CD8 T-cell levels were identified. The effect sizes of the meta-analysis of proinflammatory cytokines, including IL-6 and TNF-α, were moderate to large, while those of T cells, including CD4, were small to moderate. Other immunological indicators, such as B cells, monocytes/macrophages and immunoglobulins, might also be associated with Tourette syndrome, but further evidence is needed. Taken together, our meta-analysis consolidates the features of immune dysfunction in patients with Tourette syndrome.

Accumulated studies have deemed TS a neurodevelopmental disorder induced by dysregulated immune function, especially inflammatory responses. The TNF-α level was shown to be upregulated in 7/9 (78%) included studies, and the IL-6 level was upregulated in 6/9 (67%) studies. In this meta-analysis, TNF-α and IL-6 levels in peripheral blood were significantly increased in TS patients compared with healthy controls. However, no significant difference was identified in the expression of IL-2, IL-4, and IL-8 between TS patients and healthy controls (0 is included in the pooled SMD of these ILs). Notably, another recent meta-analysis of the immune implications in Tourette syndrome, performed by Lamothe et al. (7), only focused on ASO antibodies and anti-DNase B antibodies rather than proinflammatory cytokines. Due to the limited cytokine data, this study did not come to a conclusion about the involvement of specific cytokines in TS neurobiology.

Activation of the immune system triggered by infection is believed to be closely related to the development of TS, which has been shown in many previous studies. In contrast, a recent European multicenter study on tics in children found that GAS exposure was not associated with chronic tic disorder (CTD) (20). Interestingly, while *Mycoplasma pneumoniae* IgG positivity is not associated with a diagnosis of CTD or tic onset, it presents with a positive relationship with the severity of tic symptoms (53). This implies that differential immune status triggered by various pathogens possibly participates in different steps of TS pathogenesis.

IL-12 is a critical cytokine for immune activation, including activating natural killer (NK) cells and inducing CD4 T-cell differentiation into Th cells (54). Meanwhile, IL-12 is important for macrophage activation, inflammatory M1-type transformation and the production of macrophage-derived TNF-α and IL-6 (55, 56). Early in 2005, Leckman et al. (51) reported increased IL-12 levels in patients with TS. However, in our meta-analysis, we did not find an association of IL-12 with TS. To answer whether IL12 is implicated in the onset and progression of TS requires more bench work and more input from different studies.

Based on the results of the present study, we identified reduced total circulating CD4 T cells accompanied by an increased proportion of CD8 T cells in TS patients. Recently, a study on a TS animal model showed a reduction in rat splenic CD4 cells (57). Notably, CD4 T cells are typically divided into regulatory T (Treg) cells and conventional T helper (Th) cells. The participating role of Treg cells in TS is controversial, as one reported a reduction in the number of Treg cells and another reported an increase in activated Treg cells (58). The reduction in CD4 T helper cells could not provide confident support for the features of Treg cells, as our meta-analysis did not probe to that resolution. Instead of meta-analysis, more solid laboratory experiments need to be done to depict the role of Treg cells in TS patients.

During an infectious state, the peripheral immune system is activated, accompanied by changes in the number of T cells and increased proinflammatory cytokines. A study by Hsu et al. suggests that an activated peripheral immune system might harm the neuronal-immune system (10). Moreover, it should be noted that microglial activation might also play an important role in the neuronal-immune system of patients with TS (59). For example, a transcriptome analysis of the basal ganglia in postmortem brains from nine patients with TS indicated microglial activation in the striatum (60). Indeed, TNF-α and IL-6 have been proven to subserve the regulation of the blood–brain barrier (BBB) (61–63). Some proinflammatory cytokines may contribute to disrupting the BBB and promoting the transendothelial migration of immune cells (64, 65). With the accumulated proinflammatory cytokines crossing the BBB, microglial activation might occur in the brain (66).

Due to the limited number of studies, meta-analyses on B cells or immunoglobulins are scarce. The current study attempts to obtain information from studies on B cells and immunoglobulins. We failed to see clear effects of B cells and immunoglobulins on the development of TS. Regarding the role of monocytes/macrophages in TS, only one study with a small sample size identified increased cell numbers of monocytes/macrophages (52). More evidence is needed to explore their potential roles in immune dysfunction in TS patients. Moreover, which immune cells (including T cells, B cells or macrophages) produce elevated TNF-α and IL-6 levels in the peripheral immune system might be an important topic for future research.

Small total samples were subjected to our meta-analysis due to the limited number of available publications related to TS that enchained our analysis resolution. Hopefully, as an increasing number of TS-related publications become available, a larger sample size across different age groups could be achieved in the near future. Second, due to the limited number of included studies, a limited number of subgroup analyses or meta-regression analyses were performed to represent the heterogeneity of the meta-analysis. Last, but most importantly, the prominent heterogeneity of the data warned by our quality control step indicates that other factors could interfere with our results. For example, treatment with medicines for Tourette syndrome may change the levels of proinflammatory cytokines and T cells (67). However, due to the unavailability of the related data of the included studies, we did not explore the potential sources of the data heterogeneity, which, if we were able to accomplish, would remarkably consolidate our analysis and improve the grade of evidence.

## Conclusions

In the present meta-analysis, our results reveal increased levels of proinflammatory cytokines and deviated T-cell proportions and provide evidence for immune dysfunction in pediatric patients with TS. The proinflammatory milieu with increased IL-6 and TNF-α levels as well as reduced CD4 T helper cells is characterized. Verification of the pathophysiological roles of T cells as well as these proinflammatory cytokines in pediatric TS patients is valuable. That being said, we could still not exclude the pathogenic role of other cells, such as monocytes/macrophages or B cells, in TS due to the scarce data thus far. Furthermore, the correlation of microglial activation, which is supposed to have a direct linkage with the clinical symptoms of TS, with the immune dysregulation found in the present study is worth exploring.

## Author Contributions

For this manuscript, YiL and XW took the initiative, performed the data analysis, and completed the draft. YaL searched the included studies, and HY polished the language. YC and JG provided detailed suggestions for this study. All authors contributed to the article and approved the submitted version.

## Funding

This work is supported by the National Natural Science Foundation of China (NSFC) under Grant Nos. 82001445 and 82171538 and the Beijing Natural Science Foundation under Grant No. 7212035.

## Conflict of Interest

The authors declare that the research was conducted in the absence of any commercial or financial relationships that could be construed as a potential conflict of interest.

## Publisher’s Note

All claims expressed in this article are solely those of the authors and do not necessarily represent those of their affiliated organizations, or those of the publisher, the editors and the reviewers. Any product that may be evaluated in this article, or claim that may be made by its manufacturer, is not guaranteed or endorsed by the publisher.

## References

[1] APA: American Psychiatric Association. Diagnostic and Statistical Manual of Mental Disorders. 5th edition. Washington, DC: American Psychiatric Publishing (2013).

[2] Billnitzer A Jankovic J. Current Management of Tics and Tourette Syndrome: Behavioral, Pharmacologic, and Surgical Treatments. Neurotherapeutics (2020) 17(4):1681–93. doi: 10.1007/s13311-020-00914-6 PMC7851278 32856174

[3] Groth C Skov L Lange T Debes NM. Predictors of the Clinical Course of Tourette Syndrome: A Longitudinal Study. J Child Neurol (2019) 34(14):913–21. doi: 10.1177/0883073819867245 31411102

[4] Scahill L Specht M Page C. The Prevalence of Tic Disorders and Clinical Characteristics in Children. J Obsessive Compuls Relat Disord (2014) 3(4):394–400. doi: 10.1016/j.jocrd.2014.06.002 25436183 PMC4243175

[5] Naro A Billeri L Colucci VP Le Cause M De Domenico C Ciatto L. Brain Functional Connectivity in Chronic Tic Disorders and Gilles De La Tourette Syndrome. Prog Neurobiol (2020) 194:101884. doi: 10.1016/j.pneurobio.2020.101884 32659317

[6] Kleimaker M Takacs A Conte G Onken R Verrel J Baumer T. Increased Perception-Action Binding in Tourette Syndrome. Brain (2020) 143(6):1934–45. doi: 10.1093/brain/awaa111 32464659

[7] Lamothe H Tamouza R Hartmann A Mallet L. Immunity and Gilles De La Tourette Syndrome: A Systematic Review and Meta-Analysis of Evidence for Immune Implications in Tourette Syndrome. Eur J Neurol (2021) 28(9):3187–200. doi: 10.1111/ene.14983 34133837

[8] Domenech L Cappi C Halvorsen M. Genetic Architecture of Tourette Syndrome: Our Current Understanding. Psychol Med (2021) 51(13):2201–9. doi: 10.1017/S0033291721000234 PMC10763736 33612126

[9] Dale RC. Tics and Tourette: A Clinical, Pathophysiological and Etiological Review. Curr Opin Pediatr (2017) 29(6):665–73. doi: 10.1097/MOP.0000000000000546 28915150

[10] Hsu CJ Wong LC Lee WT. Immunological Dysfunction in Tourette Syndrome and Related Disorders. Int J Mol Sci (2021) 22(2):853. doi: 10.3390/ijms22020853 PMC7839977 33467014

[11] Ueda K Kim S Greene DJ Black KJ. Correlates and Clinical Implications of Tic Suppressibility. Curr Dev Disord Rep (2021) 8(2):112–20. doi: 10.1007/s40474-021-00230-4 PMC8224814 34178574

[12] Rizzo R Pellico A Silvestri PR Chiarotti F Cardona F. A Randomized Controlled Trial Comparing Behavioral, Educational, and Pharmacological Treatments in Youths With Chronic Tic Disorder or Tourette Syndrome. Front Psychiatry (2018) 9:100. doi: 10.3389/fpsyt.2018.00100 29636706 PMC5880916

[13] Black KJ Kim S Yang NY Greene DJ. Course of Tic Disorders Over the Lifespan. Curr Dev Disord Rep (2021) 8(2):121–32. doi: 10.1007/s40474-021-00231-3 PMC8223879 34178575

[14] Hoekstra PJ Dietrich A Edwards MJ Elamin I Martino D. Environmental Factors in Tourette Syndrome. Neurosci Biobehav Rev (2013) 37(6):1040–9. doi: 10.1016/j.neubiorev.2012.10.010 23092654

[15] Swedo SE Schrag A Gilbert R Giovannoni G Robertson MM Metcalfe C. Streptococcal Infection, Tourette Syndrome, and OCD: Is There a Connection? PANDAS: Horse or Zebra? Neurology (2010) 74(17):1397–8. doi: 10.1212/WNL.0b013e3181d8a638 20421587

[16] Martino D Chiarotti F Buttiglione M Cardona F Creti R Nardocci N. Italian Tourette Syndrome Study G: The Relationship Between Group A Streptococcal Infections and Tourette Syndrome: A Study on a Large Service-Based Cohort. Dev Med Child Neurol (2011) 53(10):951–7. doi: 10.1111/j.1469-8749.2011.04018.x 21679362

[17] Dop D Marcu IR Padureanu R Niculescu CE Padureanu V. Pediatric Autoimmune Neuropsychiatric Disorders Associated With Streptococcal Infections (Review). Exp Ther Med (2021) 21(1):94. doi: 10.3892/etm.2020.9526 33363605 PMC7725005

[18] Tsai CS Yang YH Huang KY Lee Y McIntyre RS Chen VC. Association of Tic Disorders and Enterovirus Infection: A Nationwide Population-Based Study. Med (Baltimore) (2016) 95(15):e3347. doi: 10.1097/MD.0000000000003347 PMC4839835 27082591

[19] Dehning S Matz J Riedel M Kerle IA Muller N. Symptom Exacerbation in Tourette Syndrome Due to Bacterial Reinfection. J Clin Psychiatry (2009) 70(11):1606. doi: 10.4088/JCP.08l04321whi 20031107

[20] Schrag AE Martino D Wang H Ambler G Benaroya-Milshtein N Buttiglione M. Lack of Association of Group A Streptococcal Infections and Onset of Tics: European Multicenter Tics in Children Study. Neurology (2022) 98(11):e1175–83. doi: 10.1212/WNL.0000000000013298 35110379

[21] Moller JC Tackenberg B Heinzel-Gutenbrunner M Burmester R Oertel WH Bandmann O. Immunophenotyping in Tourette Syndrome–a Pilot Study. Eur J Neurol (2008) 15(7):749–53. doi: 10.1111/j.1468-1331.2008.02159.x 18484991

[22] Quigley JM Thompson JC Halfpenny NJ Scott DA. Critical Appraisal of Nonrandomized Studies-A Review of Recommended and Commonly Used Tools. J Eval Clin Pract (2019) 25(1):44–52. doi: 10.1111/jep.12889 29484779

[23] Williams V Boylan AM Nunan D. Critical Appraisal of Qualitative Research: Necessity, Partialities and the Issue of Bias. BMJ Evid Based Med (2020) 25(1):9–11. doi: 10.1136/bmjebm-2018-111132 30862711

[24] Borenstein M Hedges LV Higgins JP Rothstein HR. A Basic Introduction to Fixed-Effect and Random-Effects Models for Meta-Analysis. Res Synth Methods (2010) 1(2):97–111. doi: 10.1002/jrsm.12 26061376

[25] Copas J Shi JQ. Meta-Analysis, Funnel Plots and Sensitivity Analysis. Biostatistics (2000) 1(3):247–62. doi: 10.1093/biostatistics/1.3.247 12933507

[26] Cohen J. Statistical Power Analysis for the Behavioral Sciences. 2nd Edition. Hillsdale, NJ: Lawrence Earlbaum Associates (1988).

[27] Balduzzi S Rucker G Schwarzer G. How to Perform a Meta-Analysis With R: A Practical Tutorial. Evid Based Ment Health (2019) 22(4):153–60. doi: 10.1136/ebmental-2019-300117 PMC10231495 31563865

[28] Yildirim Z Karabekiroglu K Yildiran A Celiksoy MH Artukoglu B Baykal S. An Examination of the Relationship Between Regulatory T Cells and Symptom Flare-Ups in Children and Adolescents Diagnosed With Chronic Tic Disorder and Tourette Syndrome. Nordic J Psychiatry (2021) 75(1):18–24. doi: 10.1080/08039488.2020.1779808 32580599

[29] Ma S-W Hou H-Y. Investigation on the Detection Value of Serum Th Cytokines and Trace Elements of Children With Tourette Syndrome. Med Innovation China (2021) 18(17):5–9.

[30] Liu H Xu X-P. Detection of Serum Sirt1 and IL-8 in Patients With Tic Disorder. Chin J Reprod Health (2020) 31(05):440–3.

[31] Hou H-J Lin S Lin X-Q Huang L-J Huang Q-Y. Changes in T Helper Lymphocytes and Their Subsets in Children With Tic Disorders. Chin J Contemp Pediatr (2018) 20(07):519–23. 10.7499/j.issn.1008-8830.2018.07.001 PMC7389206 30022750

[32] Hao L-Y. The Clinical Efficacy and Affection to Immune Cell of Jingxinzhidong Decoction for Tic Disorder [Master]. Beijing: University of Traditional Chinese Medicine (2018).

[33] Pranzatelli MR Tate ED Allison TJ. Case-Control, Exploratory Study of Cerebrospinal Fluid Chemokines/Cytokines and Lymphocyte Subsets in Childhood Tourette Syndrome With Positive Streptococcal Markers. Cytokine (2017) 96:49–53. doi: 10.1016/j.cyto.2017.03.003 28288328

[34] Lu Y Li Y-M Guo Z-P Wang X-X. Study on Immune Function of Children With Tourette Syndrome. Clin Focus (2007) 14):1022–3.

[35] Fan F Han F Wang Q. The Expressions of Serum IL-6 and TNF-α in Children With Tic Disorder. Jinagsu Med J (2017) 43(14):1005–7.

[36] Chen Y-Z Zhou K-Y Qin H-X. The Immune Function of Children With Different Clinical Types of Tic Disorder. J Clin Res (2016) 33(11):2171–2.

[37] Cheng D-J Zhang G-Y Wang J Sun Z Feng Y-Z. Changes of Levels of Serum IL-6 and TNF-α in Tic Disorder Children With Viral and Bacterial Infections. Chin J Nosocomiol (2016) 26(17):4070–2.

[38] Gao C Wu Y-L Liu J-T Wei Y-Q Xi R-Q Li X. Effects of Haloperidol Combined With Clonidine Transdermal Patch in Treatment of Children With Rerfactory Tourette Syndrome and Th1/Th2. Chin J Difficult Complicated cases (2016) 15(12):1259–62.

[39] Erzhen L Yiyan R Qian C Xiaodai C Lingyun L Ping Z. Streptococcal Infection and Immune Response in Children With Tourette's Syndrome. Child's Nerv Syst ChNS Off J Int Soc Pediatr Neurosurg (2015) 31(7):1157–63. doi: 10.1007/s00381-015-2692-8 25930720

[40] XQ Z HY L B Z ZY S. Imlmct of Xifengpinggan Heyifeiguwei Prescription On Cellular Imlnnne Function in Treating Pediatric Tourette Syndrome. China Health Stand Manage (2015) 6(29):124–6.

[41] Zhang J-Z Yang J Li E-Z Wu J-X Cui X-D Wang L-W. Functional Status of Th1 / Th2 Cells in Children With Tourette Syndrome. Chin J Psychiatry (2014) 47(02):95–8.

[42] Tang H-X Li A-Y Li J-J Hou G-S Zhang F. Effect of Ningdong Granuleon the Levels of IL-12 and TNF-α in Chlidren Patients With Tourette's Syndrome. Chin J Integr Tradit Western Med (2014) 34(04):435–8. 24812899

[43] Luo J-X Wu M Jing L-J. Relationship Between Inflammatory Cytokines and Tic Disorder of Endogenous Liver Wind Type Caused by External Wind Invasion. Acad J Shanghai Univ Tradit Chin Med (2014) 28(02):44–6.

[44] Liu Z Ji J-P Chen H Li J Zhang Y Kang J-J. Correlation Study Between T-Lymphocyte Subsets and Emotion in Children With Tourette Syndrome. China J Mod Med (2013) 23(27):101–5.

[45] Li N Du J-J Zhen H Ji W-D. Study on Relationship Between Tourette's Syndrome and ASO, IL-6, IL-8. Chin J Child Health Care (2013) 21(07):688–90.

[46] Yu-hang C Yi Z Fan H Jian-hong Y Wen-biao L Min-ling W. Detection of Autoantibodies and Increased Concentrations of Interleukins in Plasma From Patients With Tourette's Syndrome. J Mol Neurosci MN (2012) 48(1):219–24. doi: 10.1007/s12031-012-9811-8 22638859

[47] Ji J-P. Study on the Relationship Between T Cell Immunity and Related Psychosocial Factors and Tic Disorder in Children. Dalian: Dalian Medical University (2011).

[48] Gabbay V Coffey BJ Guttman LE Gottlieb L Katz Y Babb JS. A Cytokine Study in Children and Adolescents With Tourette's Disorder. Prog Neuropsychopharmacol Biol Psychiatry (2009) 33(6):967–71. doi: 10.1016/j.pnpbp.2009.05.001 PMC2770728 19427348

[49] Zhang S. Master Dissertation. The Study of Relationship Between Tourette Syndrome and Immunological Function. Jilin: Masters Diss Jilin University (2008).

[50] Mao Y-Y Master Dissertation. The Study of Cytokine IL-12 and TNF-α in Serum in Tourette Syndrome. Jilin: Masters Diss Jilin University (2007).

[51] Leckman JF Katsovich L Kawikova I Lin H Zhang H Kronig H. Increased Serum Levels of Interleukin-12 and Tumor Necrosis Factor-Alpha in Tourette's Syndrome. Biol Psychiatry (2005) 57(6):667–73. doi: 10.1016/j.biopsych.2004.12.004 15780855

[52] Matz J Krause DL Dehning S Riedel M Gruber R Schwarz MJ. Altered Monocyte Activation Markers in Tourette's Syndrome: A Case-Control Study. BMC Psychiatry (2012) 12:29. doi: 10.1186/1471-244X-12-29 22471395 PMC3356225

[53] Schnell J Bond M Moll N Weidinger E Burger B Bond R. : Mycoplasma Pneumoniae IgG Positivity is Associated With Tic Severity in Chronic Tic Disorders. Brain Behav Immun (2022) 99:281–8. doi: 10.1016/j.bbi.2021.10.012 34699932

[54] Ma X Yan W Zheng H Du Q Zhang L Ban Y. Regulation of IL-10 and IL-12 Production and Function in Macrophages and Dendritic Cells. F1000Res (2015) 4:F1000 Faculty Rev-1465. doi: 10.12688/f1000research.7010.1

[55] Kaji R Kiyoshima-Shibata J Tsujibe S Nanno M Shida K. Short Communication: Probiotic Induction of Interleukin-10 and Interleukin-12 Production by Macrophages is Modulated by Co-Stimulation With Microbial Components. J Dairy Sci (2018) 101(4):2838–41. doi: 10.3168/jds.2017-13868 29397183

[56] Huang CY Yu LC. Distinct Patterns of Interleukin-12/23 and Tumor Necrosis Factor Alpha Synthesis by Activated Macrophages are Modulated by Glucose and Colon Cancer Metabolites. Chin J Physiol (2020) 63(1):7–14. 32056981 10.4103/CJP.CJP_75_19

[57] Liu X Wang X Cao A Zhang X. Immune Function Changes of the IDPN-Induced Tourette Syndrome Rat Model. Int J Dev Neurosci (2021) 81(2):159–66. doi: 10.1002/jdn.10085 33377196

[58] Kawikova I Leckman JF Kronig H Katsovich L Bessen DE Ghebremichael M. Decreased Numbers of Regulatory T Cells Suggest Impaired Immune Tolerance in Children With Tourette Syndrome: A Preliminary Study. Biol Psychiatry (2007) 61(3):273–8. doi: 10.1016/j.biopsych.2006.06.012 16996487

[59] Frick L Pittenger C. Microglial Dysregulation in OCD, Tourette Syndrome, and PANDAS. J Immunol Res (2016) 2016:8606057. doi: 10.1155/2016/8606057 28053994 PMC5174185

[60] Lennington JB Coppola G Kataoka-Sasaki Y Fernandez TV Palejev D Li Y. Transcriptome Analysis of the Human Striatum in Tourette Syndrome. Biol Psychiatry (2016) 79(5):372–82. doi: 10.1016/j.biopsych.2014.07.018 PMC4305353 25199956

[61] Lennington JB Coppola G Kataoka-Sasaki Y Fernandez TV Palejev D Li Y. Transcriptome Analysis of the Human Striatum in Tourette Syndrome.. Biological Psychiatry (2016) 79(5):372–82. 10.1016/j.biopsych.2014.07.018 PMC4305353 25199956

[62] Chen AQ Fang Z Chen XL Yang S Zhou YF Mao L. Microglia-Derived TNF-Alpha Mediates Endothelial Necroptosis Aggravating Blood Brain-Barrier Disruption After Ischemic Stroke. Cell Death Dis (2019) 10(7):1–8. doi: 10.1038/s41419-019-1716-9 PMC6586814 31221990

[63] Fujihara K Bennett JL de Seze J Haramura M Kleiter I Weinshenker BG. Interleukin-6 in Neuromyelitis Optica Spectrum Disorder Pathophysiology. Neurol Neuroimmunol Neuroinflamm (2020) 7(5):e841. doi: 10.1212/NXI.0000000000000841 32820020 PMC7455314

[64] Sonar SA Shaikh S Joshi N Atre AN Lal G. IFN-Gamma Promotes Transendothelial Migration of CD4(+) T Cells Across the Blood-Brain Barrier. Immunol Cell Biol (2017) 95(9):843–53. doi: 10.1038/icb.2017.56 28682305

[65] Banks WA Erickson MA. The Blood-Brain Barrier and Immune Function and Dysfunction. Neurobiol Dis (2010) 37(1):26–32. doi: 10.1016/j.nbd.2009.07.031 19664708

[66] Riazi K Galic MA Kuzmiski JB Ho W Sharkey KA Pittman QJ. Microglial Activation and TNFalpha Production Mediate Altered CNS Excitability Following Peripheral Inflammation. Proc Natl Acad Sci USA (2008) 105(44):17151–6. doi: 10.1073/pnas.0806682105 PMC2579393 18955701

[67] Jones HF Han VX Patel S Gloss BS Soler N Ho A. Maternal Autoimmunity and Inflammation are Associated With Childhood Tics and Obsessive-Compulsive Disorder: Transcriptomic Data Show Common Enriched Innate Immune Pathways. Brain Behav Immun (2021) 94:308–17. doi: 10.1016/j.bbi.2020.12.035 33422639

