# Supplementary figures and images for: Profiles of Proinflammatory Cytokines and T Cells in Patients With Tourette Syndrome: A Meta-Analysis
Source: Front Immunol. 2022 May 26;13:843247. doi: 10.3389/fimmu.2022.843247 (PMC9177955; doi:10.3389/fimmu.2022.843247)

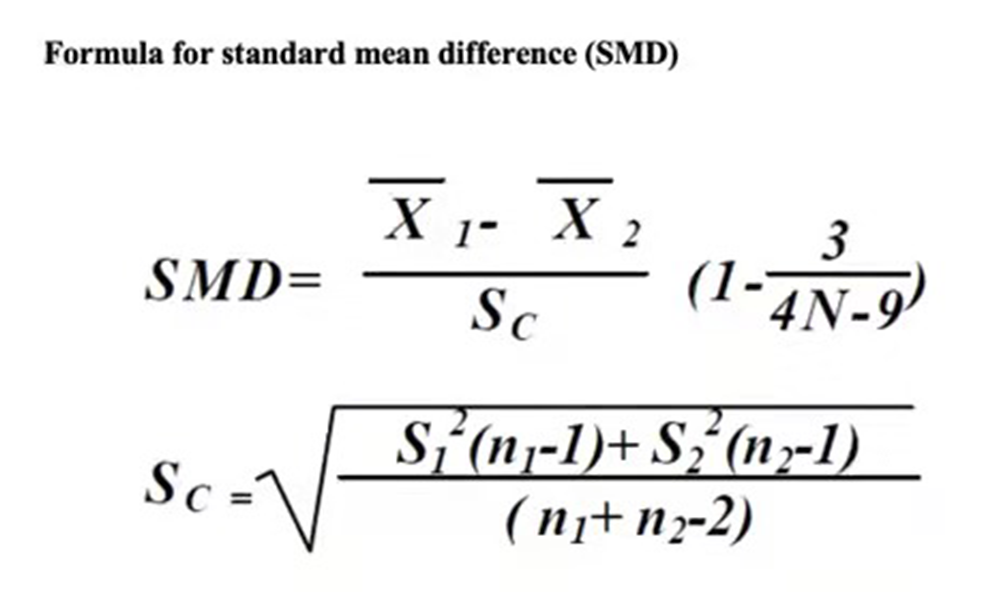

Supplement: Supplementary file 1 [file Image_1.tif]

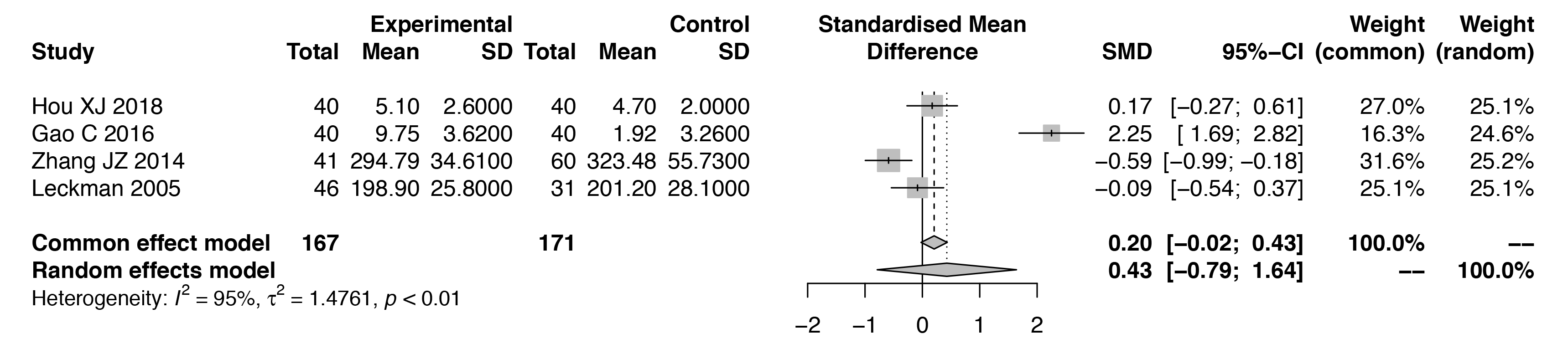

Supplement: Supplementary file 2 [file Image_2.tif]

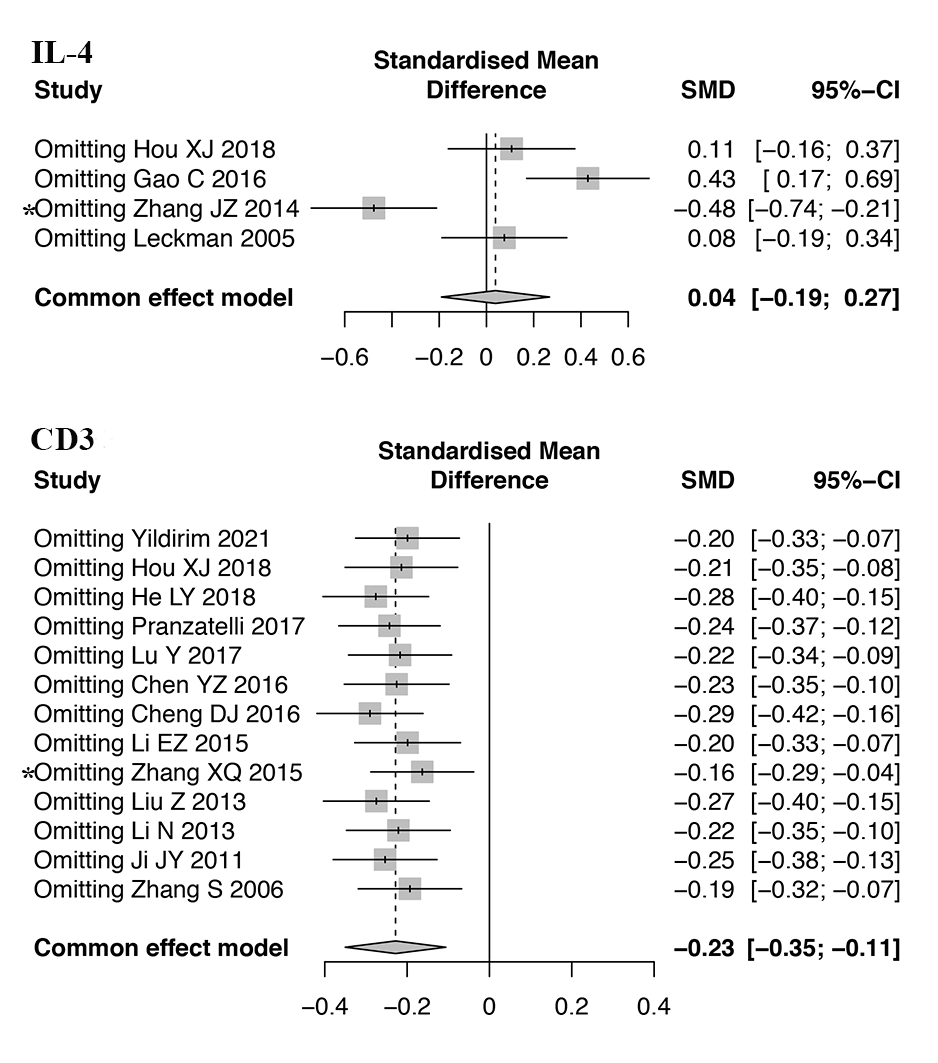

Supplement: Supplementary file 3 [file Image_3.tif]
