# Supplementary material for: Profiles of Proinflammatory Cytokines and T Cells in Patients With Tourette Syndrome: A Meta-Analysis
Source: Front Immunol. 2022 May 26;13:843247. doi: 10.3389/fimmu.2022.843247 (PMC9177955; doi:10.3389/fimmu.2022.843247)
Supplement: Supplementary file 4 [file Table_1.docx]

**Supplemental Table 1. The CASP assessment of the included studies**

| **No.** | **Author** | **Year** | **Item 1** | **Item 2** | **Item 3** | **Item 4** | **Item 5** | **Item 6a** | **Item 6b** | **Item 7** | **Item 8** | **Item 9** | **Item 10** | **Item 11** | **Number of Yes Item** |
| --- | --- | --- | --- | --- | --- | --- | --- | --- | --- | --- | --- | --- | --- | --- | --- |
| **1** | Yildirim | 2021 | **Yes** | **Yes** | **Yes** | **Yes** | **N/A** | **Yes** | **﻿Can’t Tell** | **N/A** | **N/A** | **Yes** | **﻿Can’t Tell** | **Yes** | **7** |
| **2** | Ma SW | 2021 | **Yes** | **Yes** | **Yes** | **Can’t Tell** | **N/A** | **Yes** | **Can’t Tell** | **N/A** | **N/A** | **Yes** | **Can’t Tell** | **Yes** | **6** |
| **3** | Liu HC | 2020 | **Yes** | **Yes** | **Yes** | **Yes** | **N/A** | **Yes** | **Can’t Tell** | **N/A** | **N/A** | **Yes** | **Can’t Tell** | **Yes** | **7** |
| **4** | Hou XJ^a^ | 2018 | **Yes** | **Yes** | **Yes** | **Yes** | **N/A** | **Yes** | **﻿Can’t Tell** | **N/A** | **N/A** | **Yes** | **﻿Can’t Tell** | **Yes** | **7** |
| **5** | He LY | 2018 | **Yes** | **Yes** | **Yes** | **Yes** | **N/A** | **Yes** | **Can’t Tell** | **N/A** | **N/A** | **Yes** | **Can’t Tell** | **Yes** | **7** |
| **6** | Pranzatelli | 2017 | **Yes** | **Yes** | **Yes** | **Yes** | **N/A** | **Yes** | **Can’t Tell** | **N/A** | **N/A** | **Yes** | **Can’t Tell** | **Yes** | **7** |
| **7** | Lu Y | 2017 | **Yes** | **Yes** | **Yes** | **Yes** | **N/A** | **Yes** | **﻿Can’t Tell** | **N/A** | **N/A** | **Yes** | **﻿Can’t Tell** | **Yes** | **7** |
| **8** | Fan F | 2017 | **Yes** | **Yes** | **Yes** | **Can’t Tell** | **N/A** | **Yes** | **Can’t Tell** | **N/A** | **N/A** | **Yes** | **Can’t Tell** | **Yes** | **6** |
| **9** | Chen YZ | 2016 | **Yes** | **Yes** | **Yes** | **Yes** | **N/A** | **Yes** | **Can’t Tell** | **N/A** | **N/A** | **Yes** | **Can’t Tell** | **Yes** | **7** |
| **10** | Cheng DJ | 2016 | **Yes** | **Yes** | **Yes** | **Yes** | **N/A** | **Yes** | **﻿Can’t Tell** | **N/A** | **N/A** | **Yes** | **﻿Can’t Tell** | **Can’t Tell** | **6** |
| **11** | Gao C | 2016 | **Yes** | **Yes** | **Yes** | **Yes** | **N/A** | **Yes** | **Can’t Tell** | **N/A** | **N/A** | **Yes** | **Can’t Tell** | **Yes** | **7** |
| **12** | Li EZ | 2015 | **Yes** | **Yes** | **Yes** | **Can’t Tell** | **N/A** | **Yes** | **Can’t Tell** | **N/A** | **N/A** | **Yes** | **Can’t Tell** | **Yes** | **6** |
| **13** | Zhang XQ | 2015 | **Yes** | **Yes** | **Yes** | **Yes** | **N/A** | **Yes** | **﻿Can’t Tell** | **N/A** | **N/A** | **Yes** | **﻿Can’t Tell** | **Can’t Tell** | **6** |
| **14** | Zhang JZ | 2014 | **Yes** | **Yes** | **Yes** | **Yes** | **N/A** | **Yes** | **Can’t Tell** | **N/A** | **N/A** | **Yes** | **Can’t Tell** | **Yes** | **7** |
| **15** | Luo JX | 2014 | **Yes** | **Yes** | **Yes** | **Yes** | **N/A** | **Yes** | **Can’t Tell** | **N/A** | **N/A** | **Yes** | **Can’t Tell** | **Yes** | **7** |
| **16** | Tang HX | 2014 | **Yes** | **Yes** | **Yes** | **Yes** | **N/A** | **Yes** | **﻿Can’t Tell** | **N/A** | **N/A** | **Yes** | **﻿Can’t Tell** | **Yes** | **7** |
| **17** | Li N | 2013 | **Yes** | **Yes** | **Yes** | **Can’t Tell** | **N/A** | **Yes** | **Can’t Tell** | **N/A** | **N/A** | **Yes** | **Can’t Tell** | **Yes** | **6** |
| **18** | Liu Z | 2013 | **Yes** | **Yes** | **Yes** | **Yes** | **N/A** | **Yes** | **Can’t Tell** | **N/A** | **N/A** | **Yes** | **Can’t Tell** | **Yes** | **7** |
| **19** | Cheng YH | 2012 | **Yes** | **Yes** | **Yes** | **Yes** | **N/A** | **Yes** | **﻿Can’t Tell** | **N/A** | **N/A** | **Yes** | **﻿Can’t Tell** | **Yes** | **7** |
| **20** | Ji JY | 2011 | **Yes** | **Yes** | **Yes** | **Yes** | **N/A** | **Yes** | **Can’t Tell** | **N/A** | **N/A** | **Yes** | **Can’t Tell** | **Yes** | **7** |
| **21** | Gabbay | 2009 | **Yes** | **Yes** | **Yes** | **Yes** | **N/A** | **Yes** | **Can’t Tell** | **N/A** | **N/A** | **Yes** | **Can’t Tell** | **Yes** | **7** |
| **22** | Zhang S | 2008 | **Yes** | **Yes** | **Yes** | **Yes** | **N/A** | **Yes** | **﻿Can’t Tell** | **N/A** | **N/A** | **Yes** | **﻿Can’t Tell** | **Can’t Tell** | **6** |
| **23** | Mao YY | 2008 | **Yes** | **Yes** | **Yes** | **Can’t Tell** | **N/A** | **Yes** | **Can’t Tell** | **N/A** | **N/A** | **Yes** | **Can’t Tell** | **Yes** | **6** |
| **24** | Leckman | 2005 | **Yes** | **Yes** | **Yes** | **Yes** | **N/A** | **Yes** | **Can’t Tell** | **N/A** | **N/A** | **Yes** | **Can’t Tell** | **Yes** | **7** |
| **25** | Hou XJ^b^ | 2018 | **Yes** | **Yes** | **Yes** | **Yes** | **N/A** | **Yes** | **Can’t Tell** | **N/A** | **N/A** | **Yes** | **Can’t Tell** | **Yes** | **7** |

Note: Item 1, ﻿Did the study address a clearly focused issue?; Item 2, ﻿Did the authors use an appropriate method to answer their question?; Item 3; ﻿Were the cases recruited in an acceptable way?; Item 4, ﻿Were the controls selected in an acceptable way?; Item 5, ﻿Was the exposure accurately measured to minimise bias?; Item 6a, ﻿Aside from the experimental intervention, were the groups treated equally?; Item 6b, ﻿Have the authors taken account of the potential confounding factors in the design and/or in their analysis?; Item 7, ﻿How large was the treatment effect?; Item 8, ﻿How precise was the estimate of the treatment effect?; Item 9, ﻿Do you believe the results?; Item 10, ﻿Can the results be applied to the local population?; Item 11, ﻿Do the results of this study fit with other available evidence?. Hou XJ^a^  and Hou XJ^b^ were the different sample in one study.
