# Supplementary material for: Profiles of Proinflammatory Cytokines and T Cells in Patients With Tourette Syndrome: A Meta-Analysis
Source: Front Immunol. 2022 May 26;13:843247. doi: 10.3389/fimmu.2022.843247 (PMC9177955; doi:10.3389/fimmu.2022.843247)
Supplement: Supplementary file 5 [file Table_2.docx]

**Supplemental Table 2. Other immunologic cell associated with Tourette syndrome**

| **Study** | **Experimental Group** | | |  | **Control Group** | | |
| --- | --- | --- | --- | --- | --- | --- | --- |
|  | **Total (n)** | **Mean** | **SD** |  | **Total (n)** | **Mean** | **SD** |
| **B cell** |  | **Mean (%)** | **SD (%)** |  | **Total (n)** | **Mean (%)** | **SD (%)** |
| Li EZ | 58 | 14.36 | 4.62 |  | 45 | 14.22 | 5.06 |
| Pranzatelli | 5 | 17 | 6 |  | 26 | 20 | 6 |
| **Monocytes/Macrophages** | | **Mean (cells/nL)** | **SD (cells/nL)** |  |  | **Mean (cells/nL)** | **SD (cells/nL)** |
| Matz | 46 | 0.43 | 0.27 |  | 43 | 0.37 | 0.37 |
| **IgG** |  | **Mean (g/L)** | **SD (g/L)** |  |  | **Mean (g/L)** | **SD (g/L)** |
| Lu Y | 21 | 9.66 | 1.92 |  | 30 | 10.71 | 1.66 |
| Chen YZ | 40 | 10.43 | 3.26 |  | 40 | 16.01 | 3.04 |
| Libbey | 23 | 6.34 | 3.22 |  | 25 | 5.82 | 3.35 |
| **IGA** |  | **Mean (g/L)** | **SD (g/L)** |  |  | **Mean (g/L)** | **SD (g/L)** |
| Lu Y | 21 | 1.53 | 0.52 |  | 30 | 1.27 | 0.27 |
| Chen YZ | 40 | 1.88 | 0.88 |  | 40 | 1.95 | 0.81 |
| **IgM** |  | **Mean (g/L)** | **SD (g/L)** |  |  | **Mean (g/L)** | **SD (g/L)** |
| Lu Y | 21 | 1.37 | 0.53 |  | 30 | 1.71 | 0.37 |
| Chen YZ | 40 | 1.81 | 0.72 |  | 40 | 1.74 | 0.62 |
| Libbey | 23 | 0.87 | 0.51 |  | 25 | 0.92 | 0.46 |
